# Determinants of burnout syndrome among nurses in Cameroon

**DOI:** 10.1186/s13104-018-4004-3

**Published:** 2018-12-14

**Authors:** Clarence Mbanga, Haman Makebe, Divine Tim, Steve Fonkou, Louise Toukam, Tsi Njim

**Affiliations:** 1 Mankon Sub-Divisional Hospital, Bamenda, Cameroon; 2 Regional Hospital Bamenda, Bamenda, Cameroon; 3 Clinical Research Education Networking and Consultancy, Douala, Cameroon; 4 Health and Human (2HD) Research Network, Douala, Cameroon; 5 Baptist Hospital Mutengene, Mutengene, Cameroon; 6 Faculty of Health Sciences, University of Buea, Buea, Cameroon; 7 Health Education and Research Organization (HERO), Buea, Cameroon

**Keywords:** Burnout syndrome, Nurses, Prevalence, Cameroon, Oldenburg Burnout Inventory

## Abstract

**Objectives:**

Burnout syndrome is common amongst medical personnel. The objective of this study was to identify determinants of burnout syndrome among nurses in the north west and south west regions of Cameroon.

**Results:**

A cross-sectional analysis during the months of January–June 2018 was carried out recruiting nurses consecutively after consent from state-owned and private hospitals in the English-speaking regions of Cameroon. Burnout was assessed using the Oldenburg Burnout Inventory (OLBI). Univariable regression analysis used to identify determinants of burnout syndrome among 143 nurses (mean age 29.75 ± 6.55 years) showed that being in a personal relationship (Beta = 2.25) significantly explained 3.8% of the variation in burnout (R^2^ = 3.8, F (1, 125) = 4.89, p = 0.029).

**Electronic supplementary material:**

The online version of this article (10.1186/s13104-018-4004-3) contains supplementary material, which is available to authorized users.

## Introduction

Burnout syndrome is a psychological state during which an individual experiences high levels of emotional exhaustion and disengagement from their work due to prolonged exposure to work environment stressors [1, 2]. It has been recognised by the World Health Organization (WHO) as a mental health problem and defined as a “state of vital exhaustion”; both physical and mental due to work-related stressors [3]. The prevalence of burnout syndrome amongst medical personnel ranges from 27% to 45% [4]; and is usually associated with a high prevalence of depression, suicide, use of psychoactive substances, marital problems and professional dysfunction amongst medical personnel [5, 6].

Cameroon which is one of the 57 countries described to have “critical shortages” of healthcare workers [7] has an average health personnel density of 1.3:1000; which is below the WHO recommended of 2.5:1000 [8]. The country has a physician density of approximately 1: 12,500 people [9] which rises to about 1: 26,726 persons in some regions [8]. There is a similar shortage in the health workforce in the nursing department evidenced by a nurses’ density of 0.67 per 1000 [8]. With such a high workload, nurses are at risk of having burnout syndrome in Cameroon. There is however a dearth of data in this department as with most mental health issues in Cameroon.

To bridge this gap, we set out to determine the predictors associated with the development of burnout syndrome among nurses working in the two English-speaking regions of Cameroon. This could help provide awareness on the existence of the condition and could lead to early detection. It could also help inform policymakers on the need for investigation and institution of appropriate preventive measures.

## Main text

### Methods

#### Study population

This analysis results from a mental health project which sought to identify predictors of burnout syndrome and depression amongst healthcare workers and students in Cameroon [10, 11]. Study participants were nurses working in state-owned and private institutions in the two English-speaking regions of Cameroon (North west and South west regions). The number of nurses who work in these two regions is not available. We intended to recruit all the nurses in the sample population. However, the ongoing political crises in the regions resulted in internal displacement of most healthcare providers. We were able to approach and include a total of 143 nurses via a consecutive approach.

#### Study design and protocol

A cross-sectional analysis carried out over 06 months (January–June 2018) where nurses were approached, and the purpose of the study explained. Those who agreed to participate signed a consent form. They were handed a questionnaire to fill out and return at their earliest convenience.

#### Study instrument

The data collection tool was modified from a similar study used to assess burnout and depression amongst nursing students in these regions [11]. Data collected included sociodemographic characteristics [12–14]; assessment of burnout using the Oldenberg burnout inventory (OLBI) and symptoms of depression assessed using the Patient Health Questionnaire—9 form. Responses on the OLBI were expressed on a Likert-scale from one–four corresponding to strongly agree–strongly disagree. The exhaustion and disengagement subscales of the burnout inventory were summed after reversal of negatively phrased questions to obtain a score for the OLBI.

#### Data management and statistical analysis

Collected data was entered into EPI Info version 7 for Microsoft windows, and later on exported to the Stata software package version 12 for Microsoft windows for statistical analysis.

Results obtained from the statistical analysis were presented as means (standard deviation; SD) or counts (percentages) where and when appropriate.

Predictors of burnout (assessed as a continuous variable) were determined using univariable analysis (linear regression analysis and comparisons of dependent variable against independent variables using the Kruskal–Wallis test). A multivariable analysis was to be performed if more than two variables were significant on univariable analysis. The threshold for statistical significance was set at p ≤ 0.05.

### Results

#### Sociodemographic characteristics

A total of 143 nurses were recruited. Most of them were female (96; 67.13%) (Table 1). The mean age of the participants was 29.75 ± 6.55 years (Age range; 20–55 years) (Table 2).

**Table 1:** Categorical variables showing the sociodemographic characteristics of 143 nurses in Cameroon assessed for burnout syndrome from January to March 2018

Variable	Total	
	N	%
Hospital (n = 132)		
State-owned	90	68.18
Private sector	42	31.82
Gender (n = 143)		
Male	47	32.87
Female	96	67.13
Marital status (n = 142)		
Single	73	51.41
Married	69	48.59
Personal relationship (n = 127)^a^		
Yes	78	61.42
No	49	38.58
Difficulties in personal relationship (n = 118)		
Yes	26	22.03
No	92	77.97
Majority of shifts (n = 129)		
Day	99	76.74
Night	30	23.26
Regret career choice (n = 136)		
Yes	23	16.91
No	113	83.09
Occurrence of life changing crises in last 6 months (n = 139)^b^		
Yes	57	41.01
No	82	58.99
Presence of chronic illness (n = 140)^c^		
Yes	17	12.14
No	123	87.86
Alcohol consumption (n = 141)		
Yes	71	50.35
No	70	49.65
Recreational drug use (n = 142)^d^		
Yes	7	4.93
No	135	95.07
Sufficient monthly income (n = 133)		
Yes	13	9.77
No	120	90.23

^a^Personal relationship was defined as close connections between two people formed by emotional and sexual interactions

^b^Life changing crises defined as loss of a loved one, physical or sexual trauma and condition of emotional or social instability

^c^Chronic illnesses included: Asthma, chronic pelvic pain, diabetes mellitus, gastroesophageal reflux disease, chronic peptic ulcer disease, migraines, cerebral lesions and paralysis

^d^Recreational drugs included: marijuana and tramadol

**Table 2:** Continuous variables showing the sociodemographic characteristics of 143 nurses in Cameroon assessed for burnout syndrome from January to March 2018

Variable	Number of observations	Total sample			
		Mean	SD	Min	Max
Age	108	29.75	6.55	20	55
Number of hours at work a week	127	11.50	10.80	5	90
Monthly income in USD	58	125.15	75.79	0	341.59
Number of night shifts a week	101	2.19	1.55	0	8
Quantity of alcohol consumed a week	41	1.83	1.33	0.5	6.5
Total score for disengagement items	143	17.31	3.28	8	26
Total score for exhaustion items	143	21.05	3.46	13	29
Total OLBI score	143	38.36	5.68	25	52

*USD* United states dollars, *GPA* cumulative grade point average, *OLBI* Oldenburg burnout inventory

#### Burnout syndrome

The mean OLBI score was 38.36 ± 5.68 (minimum = 25, maximum = 52) with the mean score for disengagement items being 17.31 ± 3.28 (minimum = 8, maximum = 26), and the mean score for exhaustion items being; 21.05 ± 3.46 (minimum = 13, maximum = 29) (Table 2).

#### Determinants of burnout syndrome

Determinants of burnout syndrome amongst the recruited nurses as determined by univariable analysis are shown in Table 3 and Additional file 1. Only one variable—being in a personal relationship was significant on univariable linear regression analysis, which predicted 3.8% of the variation in burnout syndrome as measured using the total OLBI score (R^2^ = 3.8, F (1, 125) = 4.89, p = 0.029). The regression equation was as follows:

$${\text{Total OLBI score }}\left( {\text{burnout syndrome}} \right)\,\,{ = }\,\, 2. 2 5\,\,\left( {\text{being in a personal relationship}} \right)\,\,{ + }\,\, 3 7. 1 4$$

**Table 3:** Univariable linear regression analysis of potential determinants of burnout syndrome amongst 143 nurses who were assessed for burnout syndrome from January to March 2018 in Cameroon

Variables	Coefficient	Intercept	95% CI	p value
Age	− 0.15	42.66	− 0.32, 0.02	0.083
Gender (female/male)	− 0.98	39.02	− 2.98, 1.02	0.334
Marital status (married/single)	− 0.39	38.56	− 2.28, 1.51	0.687
Personal relationship (yes/no)	2.31	37.04	0.28, 4.33	0.026
Difficulties in personal relationships (yes/no)	0.66	38.41	− 1.81, 3.14	0.597
Number of children	− 0.17	38.16	− 0.84, 0.50	0.615
Hospital of practice (private/state-owned)	1.22	37.86	− 0.90, 3.33	0.258
Number of hours spent in hospital	0.03	37.93	− 0.06, 0.13	0.488
Monthly income in USD	0.001	38.80	− 0.02, 0.03	0.921
Sufficient monthly income (yes/no)	0.51	38.42	− 2.87, 3.88	0.767
Majority of shifts (day/night)	− 0.38	38.55	− 2.74, 1.99	0.752
Number of night shifts a week	0.61	37.08	− 0.09, 1.31	0.085
Regret of career choice (yes/no)	0.44	38.21	− 2.11, 2.99	0.733
Recreational drug use (yes/no)	3.98	38.16	− 0.35, 8.31	0.071
Presence of chronic illness (yes/no)	− 0.97	38.56	− 3.89, 1.94	0.511
Alcohol consumption (yes/no)	0.70	37.99	− 1.20, 2.61	0.466
Quantity of alcohol consumed	0.82	37.02	− 0.72, 2.35	0.289

### Discussion

Our study was aimed at determining the determinants of burnout syndrome among nurses working in the two English-speaking regions of Cameroon. Results from statistical analysis revealed being in a personal relationship as the only significant determinant of burnout.

Our study showed that those in a personal relationship scored about two points higher in the OLBI scale than those who were not in a personal relationship. In effect, being in a relationship could come along with some degree of personal stress related to a variety of items such as the health condition of a loved one and financial strains in the relationship [15]. These stressors (though normal occurrences in a relationship) if not dealt with adequately and promptly could lead to chronic psychological stress which has been shown to be linked to poor physical and mental health outcomes [16–18]. Coupling the above-mentioned stressors resulting from personal relationships with the high workload and high levels of psychological and emotional stressors linked to the nursing profession could prove very difficult to bear. It is therefore not surprising that our study found a positive correlation between being in a personal relationship and burnout syndrome amongst the recruited nurses.

Our results correspond to those obtained by Wang et al. who found a positive relationship between work-family conflict and emotional exhaustion and cynicism amongst a group of Chinese female nurses [19]. Khamisa et al. found personal stress from relationships to be an accurate predictor of burnout among both male and female South African nurses [20]. Similar findings have been obtained elsewhere [21]. A plausible explanation to this could be the fact that nurses in a personal relationship could find themselves devoting more time and energy in trying to resolve emotional, financial and health problems within their relationships, at the expense of work-related duties. This could in turn lead to quicker exhaustion and cynicism and consequently burnout. Stressors from personal relationships have indeed been shown to interfere with work and cause higher levels of emotional exhaustion and cynicism, hence, higher levels of burnout [19, 20].

In this analysis, there was no significant differences in the total OLBI score for nurses who regretted their career choice compared to those who did not. This was contrary to what we found in another study carried out in this project where nursing students who regretted their choice in studying nursing were more likely to have higher burnout scores than those who did not [11]. This could be explained by the fact that most nurses may have already reconciled with their career choices and this will not be a significant predictor of burnout in this population.

Workload was assessed using three variables in this analysis—the number of hours spent in the hospital, the number of night shifts per week and whether most shifts worked were in the night or day. However, all three variables were not associated with burnout syndrome. This was an unexpected finding that should be investigated in future studies.

Seeking social support is seen by some as one of the most important aspect of coping with the stress associated with being in a romantic relationship [22, 23]. However, in developing settings such as ours, social support centres and professionals are far and wide apart, making it difficult for concerned individuals to access the help they need. In addition, the nursing profession is associated with long working hours and a tremendous work load. In settings such as ours where the nurses’ density is far below recommended levels [8], working hours and work load increases significantly. In the absence of the necessary professional help, having to deal with the stressors that come along with being in a relationship alone alongside the demands of the nursing profession could prove quite an uphill task. The ultimate result in such cases being quicker exhaustion and consequently burnout. In this light, some studies have found the training of nurses on coping strategies with respect to personal stress as an effective method in preventing burnout and poor mental and physical health outcomes [20, 24].

All these points to the need for more attention to be placed on the mental health status of nurses, and for the facilitation of access to professional help in case of not only work-related but also personal problems which could lead to quicker exhaustion and burnout and eventually decreased performance at work.

### Conclusion

This study could help raise awareness on burnout amongst nurses, and stir up investigations regarding appropriate preventive measures. Determinants presented in this study however require further investigations with larger sample sizes carried out nationwide, to obtain more generalizable conclusions.

## Limitations

Due to the cross-sectional nature of the study, there is the potential for recall bias in the responses of the participants and possible difficulties in ascertaining cause and effect.

Our sample size may not be large or representative enough of the nurses working in the two English-speaking regions of Cameroon as during the study period, there was an ongoing socio-political crisis in these regions of the country leading to displacement of individuals (nurses included), thereby reducing our sampling pool. There is therefore the need to carry out studies using larger sample sizes to make conclusions which could be generalised to the whole country.

However, to the best of our knowledge, this is the first multicentre study to assess the predictors of burnout syndrome amongst nurses in Cameroon and provides an invaluable insight into the predictors of burnout in this population which could guide future studies.

## Additional file

**Additional file 1.** Table showing a univariate analysis performed comparing the dependent variable—total OLBI score against the various independent variables using the Kruskal–Wallis test.

## Abbreviations

WHO: World Health Organization
OLBI: Oldenburg Burnout Inventory
