# Supplementary material for: Determinants of burnout syndrome among nurses in Cameroon
Source: BMC Res Notes. 2018 Dec 14;11:893. doi: 10.1186/s13104-018-4004-3 (PMC6295053; doi:10.1186/s13104-018-4004-3)
Supplement: Supplementary file 1 — Additional file 1. Table showing a univariate analysis performed comparing the dependent variable—total OLBI score against the various independent variables using the Kruskal–Wallis test. [file 13104_2018_4004_MOESM1_ESM.docx]

| **Variables** | **Chi-squared value** | **Degrees of freedom** | **p value** |
| --- | --- | --- | --- |
| Age | 29.86 | 25 | 0.229 |
| Gender (Female/Male) | 0.94 | 1 | 0.332 |
| Marital status (married/single) | 0.15 | 1 | 0.695 |
| Personal relationship (Yes/No) | 5.90 | 1 | 0.015 |
| Difficulties in personal relationships (Yes/No) | 0.06# | 1 | 0.802 |
| Number of children | 6.60 | 7 | 0.469 |
| Hospital of practice (Private/State-owned) | 1.04 | 1 | 0.308 |
| Number of hours spent in hospital | 13.63 | 16 | 0.626 |
| Monthly income in USD | 21.19 | 24 | 0.628 |
| Sufficient monthly income (Yes/No) | 0.27 | 1 | 0.606 |
| Majority of shifts (Day/Night) | 0.07 | 1 | 0.797 |
| Number of night shifts a week | 9.67 | 7 | 0.208 |
| Regret of career choice (Yes/No) | 0.31 | 1 | 0.58 |
| Recreational drug use (Yes/No) | 2.61 | 1 | 0.107 |
| Presence of chronic illness (Yes/No) | 0.15 | 1 | 0.704 |
| Alcohol consumption (Yes/No) | 0.84 | 1 | 0.360 |
| Quantity of alcohol consumed | 11.79 | 8 | 0.161 |
